# Egg and larval production of Eastern Baltic cod in captivity over one spawning season

**DOI:** 10.1111/jfb.70563

**Published:** 2026-07-05

**Authors:** Neele Schmidt, Marc M. Hauber, Samuel Hylander, Anssi Laurila, Felix Mittermayer, Jan Dierking

**Affiliations:** ^1^ Department of Ecology & Genetics Uppsala University Visby Sweden; ^2^ Centre for Ecology and Evolution in Microbial Model Systems (EEMiS), Department of Biology and Environmental Science Linnaeus University Kalmar Sweden; ^3^ Department of Ecology & Genetics, Evolutionary Biology Centre Uppsala University Uppsala Sweden; ^4^ GEOMAR Helmholtz Centre for Ocean Research Kiel Kiel Germany

**Keywords:** Baltic cod, egg size, spawning, thiamine

## Abstract

Eastern Baltic cod (*Gadus morhua*) has experienced a dramatic decline in biomass, distribution, size and condition over recent decades, related to a combination of fishing pressure and environmental stressors. Reduced parental size and condition are known to impact reproductive success, affecting egg quality, fertilization and hatching success and larval survival. However, how these reproductive traits vary throughout the spawning season at the population level remains poorly understood for this stock. To address this knowledge gap, this study investigated egg and larval characteristics of captive Eastern Baltic cod over an entire spawning season. Fish from three different size classes were kept in three separate tanks and allowed to spawn naturally. Eggs were collected daily from April to August, with peak spawning observed in early to mid‐June. We assessed egg and larval size, deformities, thiamine concentration, daily survival and hatching success over time. Our findings revealed high variability in these reproductive traits both across the spawning season and between size groups, without consistent temporal trends. Notably, smaller fish produced the largest eggs with the highest total thiamine concentrations, which contrasts with previous studies linking larger egg size with larger females. At the same time, these eggs showed the lowest survival and hatching rates. The only consistent pattern across all tanks was a positive relationship between egg and larval size, though unrelated to survival. These results suggest higher than expected complexity of reproductive dynamics in Eastern Baltic cod and raise the question whether the stock has undergone a shift in reproductive strategy, possibly reflecting an increased allocation towards egg quality over quantity during the recent period of stock decline.

## 1 INTRODUCTION

The Atlantic cod *Gadus morhua* (Linnaeus, 1758) stock in the Eastern Baltic Sea has experienced a severe decline in both stock biomass and distribution range over the past decades (Eero et al., 2023; ICES, 2024). This decline was accompanied by reduced body size and growth, length‐at‐maturity, a deterioration in overall condition and increased natural mortality (Casini et al., 2016; ICES, 2024; Köster et al., 2017; Neuenfeldt et al., 2020; Svedäng et al., 2022; Svedäng & Hornborg, 2017). Given the ecological and once commercial importance of this stock, understanding its reproductive biology remains of central importance.

In Atlantic cod, reproductive success is closely linked to maternal and paternal characteristics, with spawner size often associated with egg production, egg size, fertilization success, hatching success and offspring survival (Hinrichsen et al., 2016; Røjbek et al., 2014; Trippel et al., 2005). Moreover, cod are batch spawners, with individual females releasing 17–19 batches over a period of 1–2 months (Kjesbu, 1989). Reproductive traits vary considerably between batches and can show systematic trends over time. For Baltic cod in particular, previous studies have reported a decrease in egg size and an increase in egg buoyancy with batch number (Nissling et al., 1994; Vallin & Nissling, 2000). Egg size has further been correlated with key larval traits, including survival, larval length at hatch and growth (Nissling et al., 1998).

In addition to morphological egg traits, biochemical indicators may provide important information on offspring quality. Thiamine (vitamin B1) is an essential micronutrient involved in energy metabolism and early embryonic development. A deficiency in thiamine has been associated with increased mortality and behavioural abnormalities in early life stages in, for example, salmonids (Bengtsson et al., 1999; Harder et al., 2018). Given the previous indications of thiamine deficiency in Eastern Baltic cod (Engelhardt et al., 2020), egg thiamine concentration may represent an important indicator of reproductive and larval quality.

While many studies have thus addressed Baltic cod reproductive biology, most have focused on individual males and females or artificial fertilization (e.g., Åkerman et al., 1996; Biernaczyk et al., 2016; Nissling et al., 1998; Wieland et al., 1994). Consequently, our understanding of how egg and larval parameters vary over the spawning period at the population level remains limited.

To address the current knowledge gaps, we here investigated: (1) patterns in egg production, including the onset and peak of production; and (2) variations in egg and larval characteristics at population level over one spawning season. Specifically, experimental populations of male and female cod from different size classes were set up in three separate tanks, which allowed to assess population‐level reproductive patterns under standardized conditions. Individual‐level variation was not resolved. Based on current understanding, we postulate that production over the spawning season would follow a bell‐shaped curve, that size of cod would on average be positively associated with egg characteristics linked to reproductive success (e.g., higher egg size, less egg deformities, higher thiamine concentration as a potentially important quality indicator, higher daily survival and hatching success) and that these egg characteristics would in turn be positively associated with larval characteristics linking to reproductive success (e.g., larval size).

## 2 METHODS

### 2.1 Ethics statement

The care and use of experimental animals complied with the European Union Directive 2010/63/EU and the Swedish Animal Welfare Act (SFS 2018:1192), as approved by the Ethical Committee for Animal Experiments in Linköping (number 212‐2021).

### 2.2 Broodstock fish sampling and keeping

The parental cod were caught during four Baltic International Trawl Survey (BITS) cruises in February 2021, November 2021, February 2022 and March 2023 by trawling in ICES subdivision (SD) 25, corresponding to the Eastern Baltic cod stock. After capture, fish were kept on board in 0.3‐m^3^ containers with flow‐through seawater until the return to the harbour. They were then transported to the research station Ar, Gotland, Sweden, where they were kept in 15‐m^3^ tanks with recirculating seawater (7 psu, 7°C, DO: 12 mg/L, pH: 8) and a natural light cycle. The seawater in this closed system was filtered by a Recirculating‐Aquaculture‐System (RAS) with a turnover rate of 1×/h and a daily water intake of 3% to account for evaporation. On March 9th 2023, two tanks with large (total length [TL] = 61–78 cm) and medium (TL = 46–63 cm) size fish were set up, and on March 20th, one tank with small (TL = 20–41 cm) size fish was set up (Table 1). Fish in Tank 1 and Tank 2 were captured between February 2021 and February 2022 and had therefore been held in captivity for approximately 1–2 years prior to the experiment, whereas fish in Tank 3 were captured in March 2023 and had only been held for a shorter period (2 weeks) before tank setup. To prevent stress‐related impacts on the fish, sex identification was not carried out; therefore, only the total number of individuals per tank was available. After stocking each tank, the salinity in the tanks was increased gradually by adding synthetic sea salt (Hoss) until 17 psu was reached (Tank 1: March 26th, Tank 2: March 29th, Tank 3: March 31st).

**TABLE 1:** Information on broodstock in the three tanks prior to the study in March.

	Tank 1	Tank 2	Tank 3
Number of individuals	42	59	257
Total length (mean and SD)	68.5 ± 4.9 cm	54.2 ± 4.2 cm	28.8 ± 4.5 cm
Weight (mean and SD)	4482.9 ± 1105 g	2190.1 ± 606.1 g	246.6 ± 124.2 g
Fulton's K (mean and SD)	1.38 ± 0.14	1.36 ± 0.22	0.96 ± 0.13
Capture dates	February 2021–February 2022	February 2021–February 2022	March 2023

Fish were fed ca. 0.5% body weight per day with a diet consisting of frozen Atlantic herring *Clupea harengus* (Linnaeus, 1758), European sprat *Sprattus sprattus* (Linnaeus, 1758) and Northern prawn *Pandalus borealis* (Krøyer, 1838). During the spawning season, starting in April, cod were offered a lean diet of solely *P. borealis* every other day. Feeding was variable and generally discontinued during spawning. Over the experimental period, five fish died in Tank 1 before the stress episode (see below); 6 in Tank 2; and 3 in Tank 3.

Due to technical problems, on June 28th, the temperature in the RAS of Tank 1 (large cod) rose from 7 to 15°C. All fish from Tank 1 were therefore moved the next day to tanks with running water at ambient conditions (6.5–7 psu, 12–15°C). Fish were moved back to Tank 1 on July 3rd, when technical problems had been resolved and the temperature and salinity had lowered to 10°C and 9 psu, respectively. Temperature in the tank remained stable at 7°C from July 5th on, while a salinity of 17 psu was reached on July 10th and stayed stable thereafter. While the specific cause remains unclear, this episode resulted in high mortality of cod in Tank 1, with only 28 of 37 cod surviving on July 15th. Post‐mortal sex identification showed that six out of nine fish that died during this episode were female.

### 2.3 Egg sampling and measurements

From April 3rd till August 15th 2023, fertilized eggs were collected daily from the water surface in all tanks via floating airlift egg collectors (Ohs et al., 2019) and placed in separate 2‐L beakers filled with seawater (17 psu, 7°C, DO: 12 mg/L) for each tank. After approximately 10 min, when positively buoyant eggs had accumulated at the water surface and negatively buoyant eggs on the floor of the beaker, the volume of positively buoyant eggs was measured visually using the scale on the container, to the nearest 25 mL. If the total volume was <100 mL, the eggs were transferred to a 100‐mL cylinder to measure volume to the nearest mL.

From May 9th till August 1st, three replicate samples of 30 eggs each from each tank were taken for measurements once a week. All eggs were visually inspected, measured and photographed (IC Measure, version 2.0.0.286) under a stereo microscope. From the pictures, the development stage was determined according to Hall et al. (2004), and deformities were noted. Egg size was calculated as the average of three separate diameter measurements for each egg. In addition, at each timepoint, a 2‐mL sample of eggs was taken from each tank and directly frozen in an Eppendorf tube at −80°C for later thiamine content analysis.

### 2.4 Egg incubation

Between May 9th and August 1st, for each of the 13 weekly egg collections that included assessments of egg characteristics, three replicates of 100 eggs per tank were transferred and incubated in 400‐mL beakers filled with water (17 psu, 7°C, DO: 12 mg/L) prepared from filtered seawater (0.2 μm) and synthetic sea salt (Hoss). Two‐thirds of the water was changed daily and temperature, salinity and oxygen content were measured (WTW Multi 3420; temperature accuracy: ± 0.5°C, conductivity accuracy: ± 1.0% of measured value, D.O. concentration accuracy: ± 0.03 mg/L). Eggs were incubated under these conditions for a total of 14 days, by which time hatching was expected to be completed (Schmidt et al., 2024). Dead eggs and larvae, as well as egg shells were siphoned from the bottom daily and their respective numbers were noted.

### 2.5 Assessment of egg and larval characteristics

The daily number of living eggs was calculated by subtracting the total number of dead eggs from the number of eggs at the start of the incubation (*n* = 100). The number of hatched larvae was counted. Percentage of daily egg mortality and daily hatching was calculated relative to the number of eggs at the start. For sampling on 9th of May, no data on survival or hatching are available for Tank 3 due to a sampling error.

Fifteen larvae from each replicate were sedated by exposure to a 50 mg/L benzocaine solution for 10 min and then observed under a stereo microscope, photographed (IC Measure, version 2.0.0.286), assessed for deformities and measured for total length.

### 2.6 Thiamine analysis

Thiamine was extracted and quantified according to Brown et al. (1998), with modifications according to Futia et al. (2017), Futia and Rinchard (2019) and Vuorinen et al. (2002). For a detailed description, see Todisco et al. (2024). In short, around 0.5‐g cod eggs were homogenized and boiled in trichloroacetic acid. After centrifuging, the supernatant was washed with a mixture of ethyl acetate and hexane. The lower phase was mixed with a freshly made K_3_Fe(CN)_6_ solution used as the dye, filtered and then analysed by measuring fluorescence in a Hitachi Chromaster high‐performance liquid chromatography system. Three thiamine vitamers were detected: free thiamine, thiamine monophosphate and thiamine diphosphate. Vitamer concentrations were normalized per gram wet weight and summed to a total thiamine concentration (nmol/g). Two replicates were analysed for each tank from each timepoint, and a mean value was calculated. A third replicate was dried at 60°C for 48 h and weighed before and after to measure the water content and calculate total thiamine concentration per dry weight (nmol/g).

### 2.7 Statistical analysis

All statistical tests on egg and larval characteristics exclude data from Tank 1 after the stress event, from June 28th on. In figures, data from Tank 1 after the stress event are included and labelled accordingly. For visualization and analysis of positively buoyant egg volume relative to the maximal collection per tank, days with no eggs collected after the stress event in Tank 1 were excluded.

Linear mixed‐effects analysis of covariance (ANCOVA) was used to assess differences in egg and larval characteristics (i.e., egg diameter vs. larval length, survival 14 days post fertilization [DPF], and egg thiamine content, as well as egg thiamine content vs. survival 14 DPF) among tanks, to evaluate both linear and quadratic temporal trends over the spawning season, and to test for tank × time interactions. Tank was included as a fixed factor and time as a continuous covariate with both first‐ and second‐order terms. These analyses were done at replicate beaker level, which was included in the models as a random effect. Secondly, analysis of variance (ANOVA) was used to test for significance of each overall variable, that is, effect of the tank, time in spawning season and egg/larval characteristic and their interactions. When the overall mixed‐effects ANCOVA suggested divergent temporal patterns among tanks, post hoc, tank‐specific analyses were conducted. For each tank, separate linear mixed‐effects models were fitted to test linear (and where indicated, quadratic) effects of time on the parameter to confirm which tanks exhibited significant temporal trends and to characterize them as linear or non‐linear.

To test for differences in survival of eggs and larvae, that is, risk of death, between tanks (size classes) and incubation timepoints, a cox proportional‐hazards model was used. This model can be used in survival analysis when the data have a hierarchical or clustered structure (Austin, 2017). The frailty term accounting for random variation between replicate beakers contributed significantly to the model (*p* = 0.0017) and was included to ensure replication on beaker instead of individual egg level. ANOVA was used to test the significance of impact on the model of both variables.

Kruskal–Wallis rank sum test was used to test for differences in the time of first hatching, as well as time of 50% hatching. The analysis included only samples in which hatching began at 8 days post fertilization (DPF) or later, aligning with established developmental timelines for Atlantic cod (Hall et al., 2004).

To test for relations between egg and larval parameters, linear regression models including fixed effects for the parameters and their interactions were used. ANOVA further showed whether relations between parameters were significant. Outliers were removed prior to these analyses based on the interquartile range (IQR) method. For this, the IQR was multiplied by 1.5 and then this product was subtracted from the first quartile (Q1) and added to the third quartile (Q3) to define outlier boundaries.

## 3 RESULTS

### 3.1 Egg collection

**FIGURE 1:**
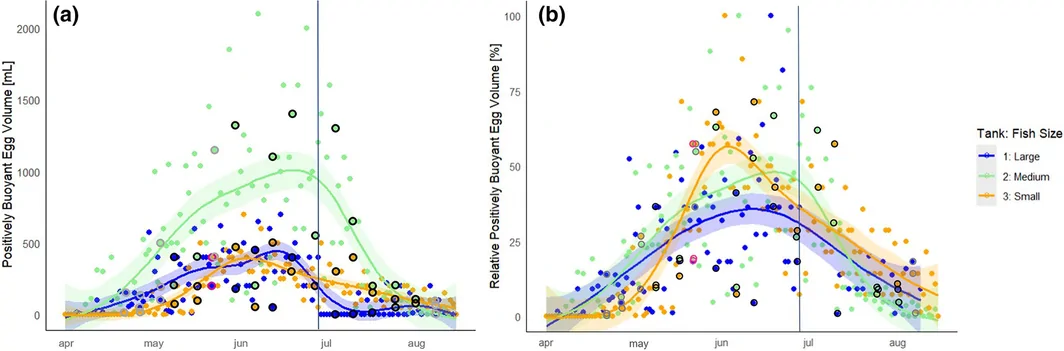
Total volume of positively buoyant eggs (a) and volume of positively buoyant eggs relative to the maximal collection per tank (b), collected daily from April to August 2023 in three tanks. Shown are generalized additive models (GAMs) for each tank, with coloured lines representing the estimated smooth trend and semi‐transparent bands representing the 95% confidence interval. Black circled indicate sampling of experimental eggs and sampling of eggs for thiamine analysis, grey circles indicate sampling of eggs for thiamine analysis only, pink circles indicate sampling of experimental eggs only. Blue vertical line indicates stress event in Tank 1.

The first positively buoyant eggs were found at the water surface in Tanks 1 and 2 on April 5th 2023, and in Tank 3 on April 19th. Overall patterns in the volume of daily egg production over the spawning season were similar for all three tanks, following roughly bell‐shaped curves. Volumes increased in all tanks from the first timepoint with collectable eggs to a peak in early to mid‐June (Tank 1: June 18th with 1100 mL; Tank 2: June 7th with 2100 mL; Tank 3: June 2nd with 700 mL) (Figures 1 and S1). From there, egg volume decreased until early to mid‐August, when collectable eggs were no longer present on a daily basis in Tank 2 and 3. Notably, although daily sampling was conducted, no eggs were successfully collected from fish of Tank 1 between the onset of the stress event on June 28th and July 11th, 1 day after the fish returned to previous temperature and salinity conditions. From then on, positively buoyant eggs were present in this tank on average only every second day until August 10th, after which date no more positively buoyant eggs were present.

### 3.2 Egg and larval characteristics over the spawning season

**FIGURE 2:**
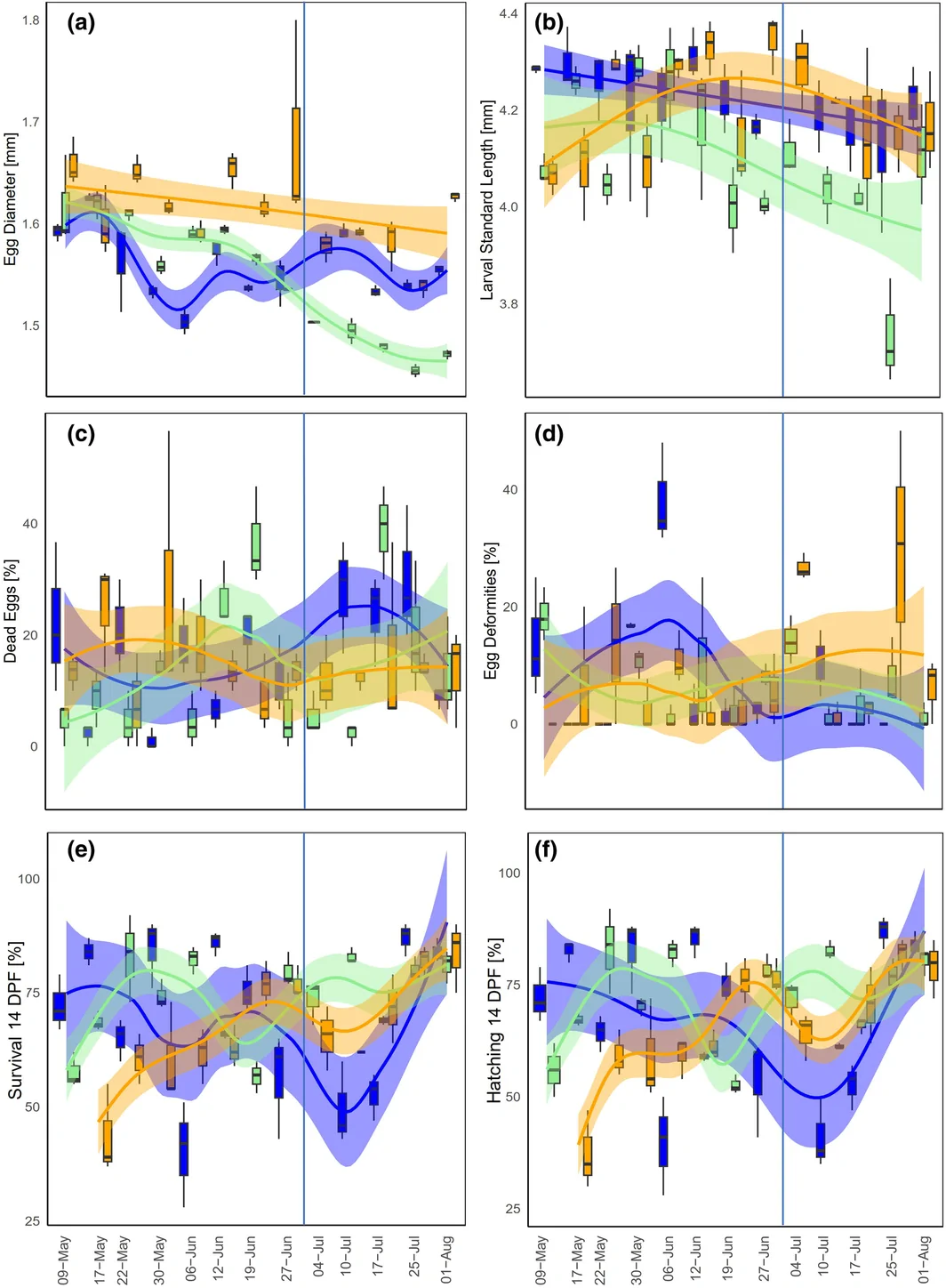
Reproductive output characteristics for cod from Tank 1 (Large cod), Tank 2 (Medium cod) and Tank 3 (Small cod); (a) egg diameter 0 DPF, (b) larval standard length 14 DPF, (c) percent of dead eggs, (d) egg deformities of living eggs 0 DPF, (e) survival of original 100 eggs 14 DPF, (f) hatching of original 100 eggs 14 DPF. Shown are GAMs for each tank, with coloured lines representing the estimated smooth trend and semi‐transparent bands representing the 95% confidence interval. Blue = Tank 1 (Large cod), green = Tank 2 (Medium cod), orange = Tank 3 (Small cod). Blue line indicates stress event in Tank 1.

Size of eggs spawned between May and August differed between the tanks (X^2^_2,102_ = 106.10, *p* ≤ 0.0001), with small‐sized fish (Tank 3) consistently producing larger eggs (1.61 ± 0.04 mm) compared to large‐ and medium‐sized fish (Tank 1: 1.56 ± 0.04 mm, and Tank 2: 1.55 ± 0.06 mm, Figure 2). There was a non‐significant trend towards smaller egg size over time from an average of 1.62 ± 0.04 (SD) mm to 1.55 ± 0.08 mm (X^2^_1,102_ = 3.48, *p* = 0.06), with no significant difference in patterns among tanks (interaction term tank and time, X^2^_2,102_ = 5.25, *p* = 0.07).

In contrast, there were significant differences in larval length between tanks (X^2^_2,102_ = 26.93, *p* ≤ 0.0001), with large fish producing larger larvae than medium‐sized fish, that is, 4.25 ± 0.09 mm versus 4.09 ± 0.16 mm (Welch's *t*‐test: t_61_ = −4.39, *p* < 0.001). Larvae from small‐sized fish (4.20 ± 0.12 mm) were also significantly larger than from medium‐sized fish (Welch's *t*‐test: t_76_ = −3.49, *p* < 0.001) and post hoc analysis showed a curvilinear pattern, as both the linear (X^2^_1,39_ = 13.59, *p* ≤ 0.0001) and quadratic (X^2^_1,39_ = 12.72, *p* = 0.001) component in length over time were significant. Contrary to this, medium fish showed a linear decline in larval length over the spawning season (X^2^_1,39_ = 12.55, *p* < 0.001), based on a linear‐only post hoc model as adding the quadratic term did not improve model fit (maximum likelihood‐based likelihood‐ratio test: X^2^_1,39_ = 1.10, *p* = 0.29; Akaike information criterion [AIC]: −37.90 vs. −37.00).

Neither the amount of dead eggs nor egg deformities showed consistent differences between the tanks (X^2^_2,102_ = 0.27, *p* = 0.87, and X^2^_2,102_ = 1.94, *p* = 0.38, respectively). On average, dead eggs accounted for 7.68 ± 10.6% of collected eggs, and deformities comprised 14.35 ± 11.49% of living eggs across the tanks. Further, no differences in the amount of dead eggs (linear: X^2^_1,102_ = 0.19, *p* = 0.66 and non‐linear: X^2^_1,102_ = 0.14, *p* = 0.71) or egg deformities (linear: X^2^_1,102_ = 0.28, *p* = 0.60 and non‐linear: X^2^_1,102_ = 0.04, *p* = 0.84) were present over time.

Overall survival at 14 DPF as well as hatching 14 DPF showed high fluctuations and followed similar patterns over the spawning season, averaging at 70.50 ± 16.00%, 73.46 ± 9.26% and 67.33 ± 11.38% in survival, and at 69.46 ± 16.45%, 71.31 ± 10.79% and 65.47 ± 13.01% in hatching for Tank 1, Tank 2 and Tank 3, respectively. Consequently, the great majority of surviving eggs successfully hatched at the end of the experiment. Both variables differ between tanks (survival: X^2^_2,99_ = 7.78, *p* = 0.02, hatching: X^2^_2,99_ = 7.73, *p* = 0.02), with offspring from small‐sized fish showing lowest survival and hatching. Neither differences in survival (linear: X^2^_1,99_ = 1.56, *p* = 0.21 and non‐linear: X^2^_1,99_ = 0.36, *p* = 0.55), nor hatching over time (*F*_12,18_ = 2.37, *p* = 0.01) were detected in the overall model. Post hoc analysis however showed a linear increase in survival (X^2^_1,36_ = 3.85, *p* = 0.05) and hatching over time (X^2^_1,36_ = 7.62, *p* ≤ 0.01) for small‐sized fish.

A Cox proportional‐hazards model further showed that the timing of mortality events for cod eggs and larvae did not only differ between tanks (X^2^_2_ = 39.71, *p* ≤ 0.001), but in contrast to final survival 14 DPF also between timepoints in the spawning season (X^2^_12_ = 162.70, *p* ≤ 0.001; Figure S2), with an overall increase in survival chance over time. While survival chance was similar in Tank 1 and 2 (hazard ratio (HR) = 0.97, 95% confidence interval (CI): 0.88–1.07, *p* = 0.57), survival chance was lower in Tank 3 (HR = 1.32, 95% CI: 1.19–1.46, *p* ≤ 0.0001). The likelihood‐ratio test for the model (*n* = 9900, number of events = 2911) using tank as a fixed variable, incubation timepoint as a covariate and replicate as a random effect (39 groups, X^2^_0_ = 0.002, *p* = 0.0017) was highly significant (X^2^_14_ = 202.4, *p* < 0.001) and the concordance index was 0.581 (SE = 0.005).

**FIGURE 3:**
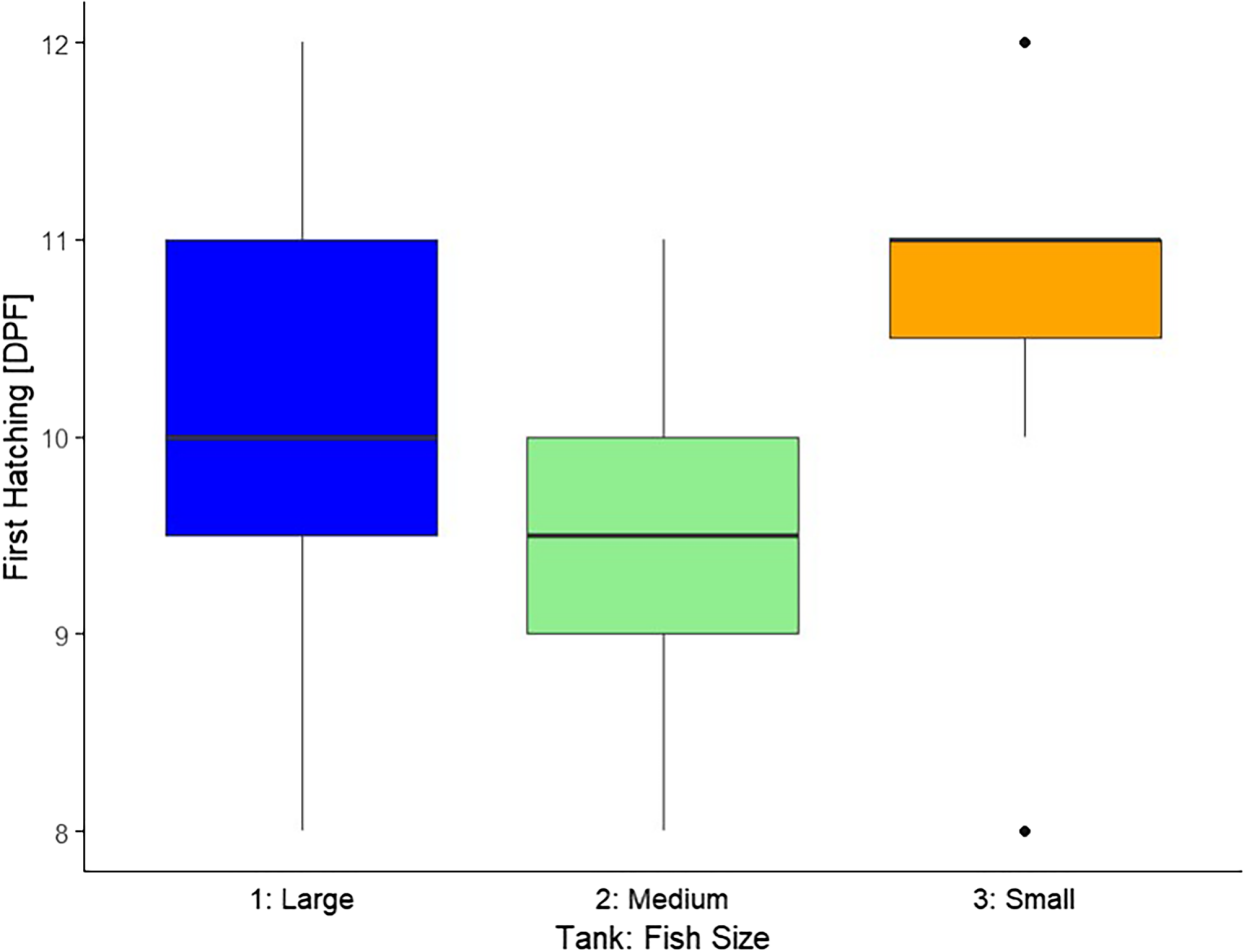
Time of first hatching of Baltic cod larvae in days post fertilization [DPF] from the three different tanks. Shown are data aggregated from all incubation timepoints during the spawning season, excluding post stress event data for Tank 1 (Large cod). Blue = Tank 1 (Large cod), green = Tank 2 (Medium cod), orange = Tank 3 (Small cod).

First hatching differed between tanks (X^2^_2_ = 24.91, *p* ≤ 0.001; *n* = 86; Figure S2, Figure 3), as larvae from Tank 2 started to hatch earliest, at 9.5 DPF (9–10; median IQR), while hatching of larvae from Tank 1 started 10 DPF (9.5–11), and began latest for larvae from Tank 3, at 11 DPF (10–11). At 13 DPF, ≤50% of larvae had hatched in all three tanks (X^2^_2_ = 1.73, *p* = 0.42; *n* = 99; IQR Tank 1: 13–13, Tank 2: 12.5–13, Tank 3: 13–13).

**FIGURE 4:**
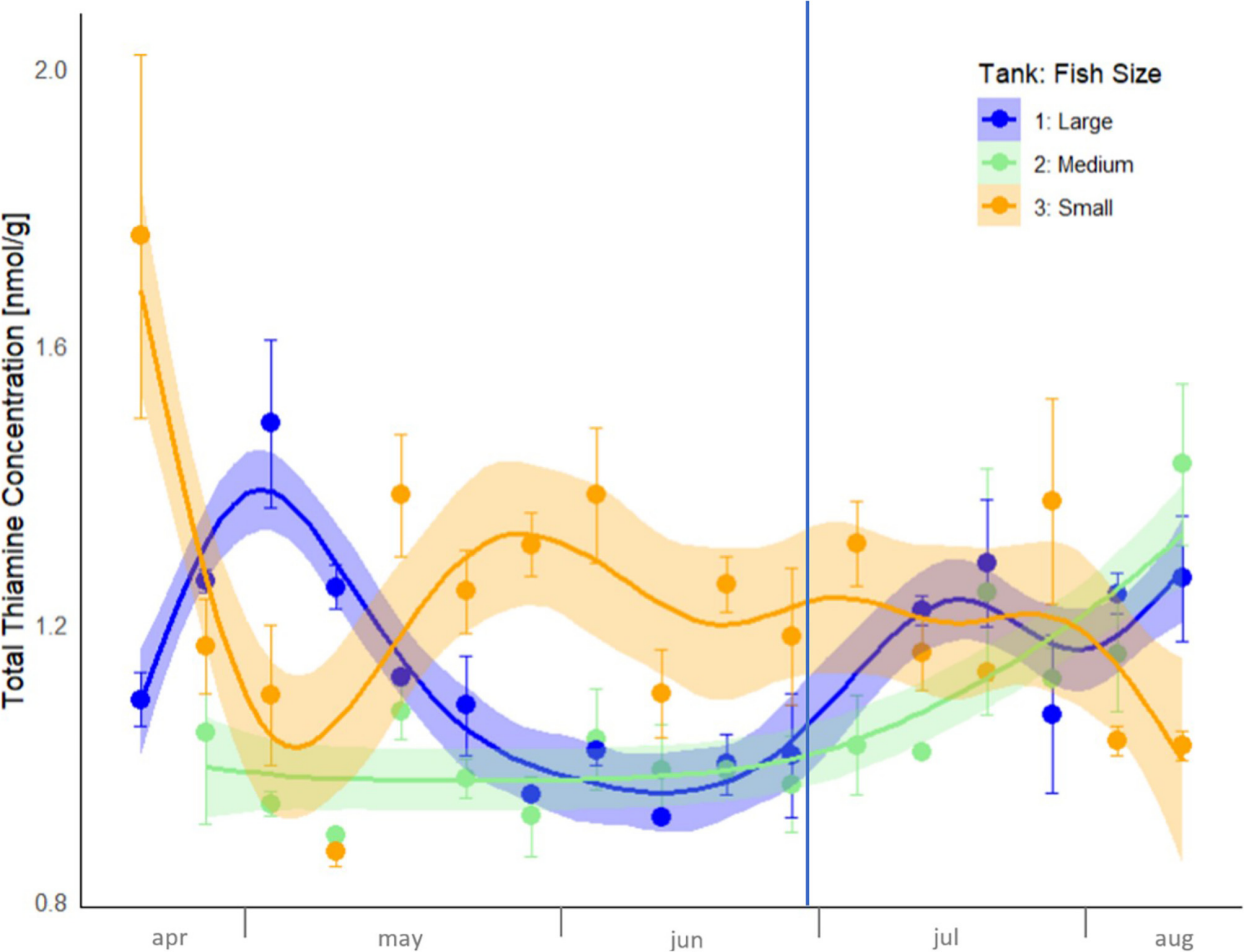
Egg thiamine concentration per wet weight 0 days post fertilization (DPF) in the three different tanks over the spawning season. Shown are generalized additive models (GAMs) for each tank, with coloured lines representing the estimated smooth trend and semi‐transparent bands representing the 95% confidence interval. Blue line indicates stress event in Tank 1.

Total thiamine concentrations per g wet weight in cod eggs 0 DPF mainly ranged between 1.0 and 1.4 nmol/g during the spawning season from April to August (Figure 4) and differed between tanks (X^2^_2,88_ = 22.65, *p* ≤ 0.001). Eggs from small‐sized fish had higher concentrations than eggs from medium or large fish (Welch's *t*‐test: t_58_ = −4.20, *p* < 0.001 and t_50_ = −2.37, *p* = 0.02, respectively), which were of similar concentration (Welch's *t*‐test: t_39_ = 1.37, *p* = 0.18). No significant differences were detected between timepoints (linear: X^2^_1,88_ = 3.51, *p* = 0.06, non‐linear: X^2^_1,88_ = 3.14, *p* = 0.08) or in tank and time interactions (linear: X^2^_2,88_ = 0.76, *p* = 0.68, non‐linear: X^2^_2,88_ = 3.67, *p* = 0.16). Total thiamine concentrations per dry weight mainly ranged between 25 and 40 mg/g (see Figure S3) and overall patterns were similar to wet weight fluctuations.

### 3.3 Relations between characteristics

**FIGURE 5:**
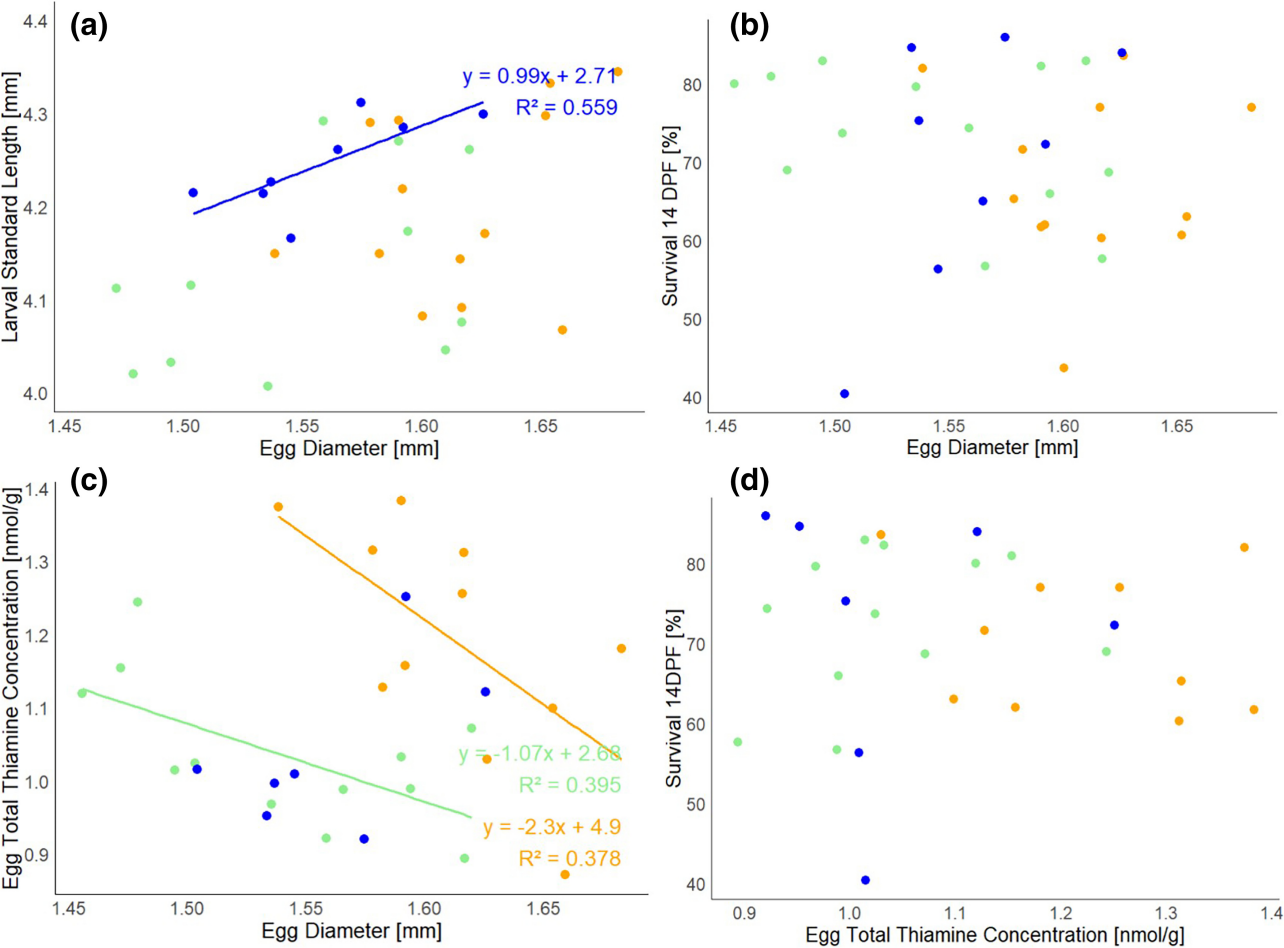
Relation between (a) egg diameter and larval length, as well as (b) egg diameter and total thiamine concentration 0 DPF, (c) egg diameter and survival 14 DPF and (d) egg total thiamine concentration and survival 14 DPF. Blue = Tank 1 (Large cod), green = Tank 2 (Medium cod), orange = Tank 3 (Small cod).

Larval length showed a significant positive relation to egg size (*F*_1,27_ = 4.48, *p* = 0.04; Figure 5). This relationship (interaction term) was consistent among sizes (*F*_2,27_ = 0.04, *p* = 0.96), but only significant in large fish (*F*_1,6_ = 6.52, *p* = 0.03), where it accounted for 55.9% of larval length variance. No interaction between egg size and tank (*F*_2,27_ = 3.13, *p* = 0.06) and no overall effect of tank (*F*_2,27_ = 0.64, *p* = 0.54) nor egg size (*F*_1,27_ = 0.003, *p* = 0.95) on survival 14 DPF was detected.

Further, a relationship between egg size and thiamine concentration (*F*_1,24_ = 5.72, *p* = 0.02) was found. However, the relationship differed between tanks (*F*_2,24_ = 4.20, *p* = 0.03), with egg size negatively correlating with thiamine concentration in medium and small fish (*F*_1,10_ = 6.54, *p* = 0.03 and *F*_1,9_ = 5.46, *p* = 0.04, respectively), whereas large fish showed a positive but non‐significant slope (*F*_1,5_ = 2.27, *p* = 0.19). No significant effect of thiamine concentration (*F*_1,23_ = 0.44, *p* = 0.65) on survival 14 DPF was detected.

### 3.4 Stress effect

Fish in Tank 1 experienced stress, first from the temperature increase, then also from the placement in other tank systems with less controlled water parameters and lower salinity, from 29th June to 3rd July. After being moved back, salinity in the tank had to be gradually increased again, reaching 17 psu by July 10th. Collectable eggs were observed at the water surface the day after. Notably, both the volume of positively buoyant eggs and the spawning periodicity changed substantially compared to pre‐stress event observations—eggs were now produced every other day and in reduced volume. Nevertheless, key reproductive characteristics showed no significant differences when averaged 3 weeks prior and 3 weeks after the event except for an increase in the amount of dead eggs per collection of about 14%, and a small increase in total thiamine concentration of 0.21 nmol/g (Table 2).

**TABLE 2:** Key reproductive characteristics from incubations 3 weeks before the stress event and 3 weeks after egg collection resumed in the tank.

	Before stress event	After stress event	Welch's *t*‐test	*p* value
Egg diameter	1.59 ± 0.05 mm	1.56 ± 0.05 mm	*t* = 1.22, df = 13.59	0.24
Dead eggs	12.96 ± 7.54%	27.14 ± 9.33%	*t* = −3.55, df = 15.33	0.003^*^
Egg deformities	2.06 ± 2.62%	3.48 ± 5.92%	*t* = −0.66, df = 11.01	0.52
Egg thiamine	0.98 ± 0.06 mg/g	1.19 ± 0.12 nmol/g	*t* = −3.94, df = 7.53	0.005^**^
Hatching	71.33 ± 15.31%	60.22 ± 21.32%	*t* = 1.27, df = 14.52	0.22
Survival	72.56 ± 14.56%	63.11 ± 18.79%	*t* = 1.19, df = 15.01	0.25
Larval length	4.23 ± 0.08 mm	4.16 ± 0.10 mm	*t* = 1.71, df = 15.09	0.11

*Note*: Shown are mean values along with standard deviation. Note that incubations started again 1 week after the stress event, that is, after fish have been back in Tank 1 for 1 week.

* *p* < 0.05.

** *p* < 0.01.

## 4 DISCUSSION

The main finding of this study is that egg and larval characteristics of captive Eastern Baltic cod varied markedly over the spawning season, but without consistent seasonal trends and linkage to fish size across most traits that we would have expected based on published literature. While larval size increased with egg size, unexpectedly, smaller fish produced larger eggs, underscoring the complexity of reproductive dynamics at the population level. These findings provide rare population‐level insights from controlled tank experiments, improving our understanding of reproductive dynamics in a species facing multiple environmental and anthropogenic stressors and population decline.

### 4.1 Egg production throughout the spawning season

The temporal patterns in spawning observed in this study, with the onset in April, cessation in August and peak spawning in June, closely reflect the current natural spawning phenology of Eastern Baltic cod in the wild, with spawning mainly from May to August and highest egg abundances in June (ICES, 2019). Historically, however, during periods of high population abundance, the spawning period of Eastern Baltic cod was longer, starting as early as March and lasting till October–November (Bagge et al., 1994; Baranova et al., 2011), linking to a more diverse age and maturity structure within the stock, as well as to favourable and stable environmental conditions that supported reproductive activity across several months (Karasiova et al., 2008).

In this study, spawning in the tank with smaller fish began later than in tanks with larger individuals, consistent with previous findings by Tomkiewicz and Köster (1999). However, contrary to their report of no size‐related differences in peak spawning, we observed an earlier and concentrated spawning peak in the smaller fish. This suggests a higher synchronization in reproductive activity among smaller individuals, whereas larger fish spread spawning more evenly throughout the season.

### 4.2 Patterns in egg characteristics

Patterns in egg size over the spawning season were less conclusive. The borderline significant trend towards smaller egg size was generally in line with the decline previously reported by Vallin and Nissling (2000), but substantially weaker (1.62 ± 0.04 (SD) mm to 1.55 ± 0.08 mm in our study versus 1.62 ± 0.07 mm to 1.40–1.43 mm for fish in a similar size range). Declining egg size as spawning progresses has been associated with the depletion of maternal energy reserves for batch spawners (Mcevoy & Mcevoy, 1991; McEvoy & McEvoy, 1992; Trippel & Neil, 2004), as well as with smaller females typically spawning later and producing smaller eggs compared to larger females (Tomkiewicz & Köster, 1999; Vallin & Nissling, 2000). However, interpretation of egg diameter patterns in Baltic cod should be approached with caution, as final oocyte maturation is accompanied by substantial water uptake and egg swelling (Nissling et al., 1994; Thorsen et al., 1996), and incomplete eggshell hardening after spawning may lead to artificially inflated egg diameters (Oppen‐Berntsen et al., 1990). In our study, the available dry‐weight estimates generally followed the same temporal patterns as wet egg size, suggesting that swelling effects were relatively consistent throughout the spawning season, although the limited number of dry‐weight measurements precluded a more robust analysis. Notably, in this study the fish were grouped by size classes, but individuals within each tank still varied substantially in size, by up to 20 cm. Consequently, the subtle decline in egg sizes over time observed in this study may be linked to this size variation, as larger individuals may have spawned earlier than smaller ones within the same tank.

Aside from this trend, clear seasonal patterns in other egg characteristics were not apparent. Only the small fish showed increasing survival and hatching over time, a pattern that may be an artefact of their recent transfer from the wild and late establishment of their tank, in contrast to longer‐held medium and large fish. The lack of consistent patterns across other traits implies that other factors of individual variation among females, for example, genetics, previous spawning effort, stress levels, nutritional status or mate choice, may have affected offspring characteristics (Trippel et al., 2005). As possibly multiple females contributed to egg batches collected at each timepoint, this variability may have masked any subtle seasonal trend.

Unexpectedly, the largest eggs were produced in the tank with the smallest fish, contrasting with the many studies reporting that larger and older Baltic cod produce larger eggs (e.g., Kjesbu, 1989; Nissling & Vallin, 1996; Vallin & Nissling, 2000). Additionally, several fish in this tank are likely first‐time spawners, given their average length of 28 cm, which is near the reported size at 50% maturity (Svedäng et al., 2024). Contrary to our results, first‐time spawning cod are typically found to produce smaller eggs, yet the observed lower survival and hatching success compared to larger repeat‐spawners align with previous studies (Solemdal et al., 1995; Trippel, 1998). Beyond spawning experience, egg size is influenced by parental condition (Chambers & Waiwood, 1996; Mion et al., 2018), which is inseparable from size in this study. Given the concurrent declines in both size and condition of Baltic cod in the wild (Casini et al., 2016; Svedäng et al., 2024), further investigations into whether this aligns with increased egg sizes in natural conditions are needed.

In the wild, Baltic cod have increasingly been exposed to stressful conditions such as rising temperatures and expanding hypoxic zones (Casini et al., 2016; Dutheil et al., 2022; Reusch et al., 2018). Importantly, larger eggs offer potential adaptive benefits under such conditions, as they maintain neutral buoyancy in lower salinities, which enables development in more favourable oxygen conditions (Vallin & Nissling, 2000), and were associated with higher thiamine concentrations, essential for several central cellular processes (Kraft & Angert, 2017). Notably, small cod had less time to acclimatize to captivity and the facility's feed, that is composed similarly to their natural diet, but of sufficient quantity. However, the higher thiamine concentrations in their eggs are consistent with previous observations that smaller cod from the Baltic Proper have higher gonadal thiamine concentrations than larger specimens from, for example, the North Sea or Åland Sea (Hauber et al., 2026). Hence, we believe that natural processes rather than the shorter acclimatization period to captivity have produced these patterns. Overall, the trade‐off between producing larger but fewer versus smaller but more eggs has long been recognized (Einum & Fleming, 2000; Stearns, 1992), as larger offspring size has been shown beneficial in suboptimal or highly variable environments (Elgar, 1990; Parker & Begon, 1986; Reznick, 1991; Roff, 1992). This raises the question whether smaller cod may have adapted a compensatory strategy, investing in larger but fewer eggs to offset higher offspring mortality.

### 4.3 Links between egg and larval characteristics

The only consistent pattern that was in line with expectations prior to carrying out this study was the positive correlation between egg size and larval length. This finding was in line with prior results by Nissling et al. (1998), who linked not only larval length at hatch, but also post‐hatch growth and viable hatch to egg size in cod. Smaller larvae are not only more vulnerable to early mortality due to limited endogenous energy reserves, but larval size at hatch in cod has also been closely associated with several key developmental traits, such as feeding incidence, the timing of point of no return, development of a swim bladder and growth beyond the yolk‐sac stage (Knutsen & Tilseth, 1985; Marteinsdottir & Steinarsson, 1998). However, unlike Nissling et al. (1998), we did not detect a direct effect of egg size on survival, nor between survival and egg thiamine concentration, despite correlations reported in other species (Fisher et al., 1998). These earlier findings were based on individual females, whereas our study examined pooled data from captive populations. The highly complex and variable patterns observed in this study suggest that, in population‐level settings, traits like survival may be more strongly influenced and in part buffered by other factors, making consistent patterns harder to detect.

### 4.4 Thiamine concentrations

Few studies have investigated thiamine (deficiency) in cod (Amcoff et al., 1999, Engelhardt et al., 2020; for an in‐depth discussion, see Hauber et al., 2026). To our knowledge, this study is the first to describe thiamine concentrations of cod eggs post spawning. Comparing these values to published literature on thiamine concentrations in gonadal tissues/ developing eggs ranging from 3 to 90 nmol/g, we see overall lower concentrations in post‐spawning eggs (Hauber et al., 2026). This difference/reduction in concentrations is likely due to the hydration/swelling process once they were released into the water column. Even closely related fish species can vary in their thiamine concentrations and thresholds (see Harder et al., 2018 for more information). Cod life history differs significantly, especially reproductive and early life, from, for example, salmonids, that are more exensivley studied in terms of thiamine deficiency. Hence, applying known thresholds for thiamine deficiency in salmonids on cod is questionable. However, thiamine concentrations did not show any relationship with hatching/survival success. This indicates that the measured levels are sufficient for larval development before external feeding starts. Since healthy eggs were produced by fish that were only recently transferred to captivity and had little time stocking up on the facility's feed, we conclude that thiamine was not a limitating factor to cod reproduction in the facility and it might even not currently be limiting to fish in the wild. This would align with results by Hauber et al. (2026), who found no current signs of thiamine deficiency in Baltic cod stocks.

### 4.5 Recovery after the stress event

Apart from a slight increase in dead eggs and egg thiamine concentration, none of the measured egg and larval characteristics showed a significant change following the stress event, suggesting a surprisingly rapid recovery after substantial environmental fluctuations that would be in line with a moderate heat wave and strong freshwater inflow. The absence of collectable eggs at the water surface following the stress event in the alternative tank could be due to Eastern Baltic cod eggs being neutrally buoyant at 14.5 psu (Nissling et al., 1994; Nissling & Westin, 1991), that is, eggs not being positively buoyant until salinity in Tank 1 was back to previous conditions. However, the spawning frequency and volume noticeably decreased, possibly indicating that after accounting for mortality of fish in the tank, only few females were left. During the final week of sampling, egg production ceased in the tank, which may reflect either the natural end of the spawning period for the remaining females, or the early conclusion of spawning due to stress. Overall, the continuation of successful spawning activity after the stress event would suggest capacity to buffer environmental perturbations during the spawning period in the wild.

### 4.6 Limitations of this study

The fish populations in the three study tanks differed regarding number of individuals, likely spawning experience, and time in captivity, with larger fish having been held at the facility for a longer period. Such differences in acclimatization to captivity have previously been shown to impact egg and larval characteristics (Nissling et al., 1998). Additionally, carry‐over effects from prior in vivo environmental conditions before capture, particularly lower salinity than in captivity, may have further influenced reproductive traits. Specifically, egg size is closely linked to egg hydration and resulting buoyancy, as egg density is regulated through oocyte hydration within the ovary of the maternal fish (Nissling et al., 2003) to match the ambient salinity conditions experienced by the spawner (Goarant et al., 2007). Therefore, differences in prior salinity exposure may have contributed to the observed variation in egg size among tanks. These effects may also extend to, for example, hatching success, survival‐related results and larval size.

Moreover, potential variation in female‐to‐male ratios between tanks may have influenced reproductive dynamics and output. Consequently, a tank effect cannot be excluded and differences observed in egg and larval characteristics between tanks in this study may not be exclusively linked to parental size. This limitation is particularly relevant for comparisons among size classes, whereas such effects, if present, would be unlikely to affect relative patterns over time.

## 5 CONCLUSION

This study demonstrates the complexity of seasonal pattern in egg and larval characteristics of captive Eastern Baltic cod populations. While our results showed the expected bell‐shaped curve in egg production over the spawning season, we found high variability within other traits over time and between tanks, contrary to our hypothesis of consistent patterns. The single consistent relationship that was found across all tanks was an increase in larval size with increasing egg size, which is typically associated with higher survival, though unrelated in this study. Unexpectedly, larger eggs were produced by smaller fish, contrasting the many previous studies showing larger fish to produce larger eggs. These findings highlight the need for further research into whether smaller Baltic cod nowadays may be allocating more energy resource into the quality (size) over quantity of eggs produced as a sign of adaptation to environmental stress.

## AUTHOR CONTRIBUTIONS

N.S.: conceptualization, methodology, investigation, data curation, formal analysis, visualization, writing – original draft, M. M. H.: conceptualization, methodology, investigation, writing – section, review and editing, S. H.: conceptualization, funding acquisition, writing – section, review and editing, F.M.: validation, writing – review and editing, A. L.: funding acquisition, validation, writing – review and editing, J. D.: formal analysis, validation, writing – review and editing.

## FUNDING INFORMATION

This work was conducted as part of the collaborative project ReCod funded by the foundation BalticWaters and the faculty of science and technology at Uppsala University. Additional funding was received from VR (grant no.: 2019‐04251) and FORMAS (2020‐01514) to S.H.

## Supporting information


**Figure S1.** Total volume of positively buoyant eggs standardized per total biomass, collected daily from April to August 2023 in three tanks. Shown are generalized additive models (GAMs) for each tank, with coloured lines representing the estimated smooth trend and semi‐transparent bands representing the 95% confidence interval. Blue vertical line indicates stress event in Tank 1.
**Figure S2.** Daily survival and hatching of Baltic cod eggs and larvae from the three different tanks over time. Shown are mean values of the three replicates for different timepoints during the spawning season. Blue = Tank 1 (Large cod), green = Tank 2 (Medium cod), orange = Tank 3 (Small cod). Dotted and dashed lines for Tank 1 show data collected after the stress event.
**Figure S3.** Egg thiamine concentration per dry weight 0 days post fertilization (DPF) in the three different tanks over the spawning season. Shown are generalized additive models (GAMs) for each tank, with coloured lines representing the estimated smooth trend and semi‐transparent bands representing the 95% confidence interval. Blue line indicates stress event in Tank 1.

## Data Availability

The data that support the findings of this study are openly available in Figshare at https://doi.org/10.6084/m9.figshare.30344077.
