# Supplementary material for: Egg and larval production of Eastern Baltic cod in captivity over one spawning season
Source: J Fish Biol. 2026 Jul 5;109(3):1844–55. doi: 10.1111/jfb.70563 (PMC13602103; doi:10.1111/jfb.70563)
Supplement: Supplementary file 1 [file JFB-109-1844-s001.docx]

**Supplementary**

Figure S1: Total volume of positively buoyant eggs standardized per total biomass, collected daily from April to August 2023 in three tanks. Shown are Generalized Additive Models (GAMs) for each tank, with coloured lines representing the estimated smooth trend and semi-transparent bands representing the 95% confidence interval. Blue vertical line indicates stress event in Tank 1.

Figure S2: Daily survival and hatching of Baltic cod eggs and larvae from the three different tanks over time. Shown are mean values of the three replicates for different timepoints during the spawning season. Blue = Tank 1 (Large cod), green = Tank 2 (Medium cod), orange = Tank 3 (Small cod). Dotted and dashed lines for Tank 1 show data collected after the stress event.

Figure S3: Egg thiamine concentration per dry weight 0 days post fertilization (0 DPF) in the three different tanks over the spawning season. Shown are Generalized Additive Models (GAMs) for each tank, with coloured lines representing the estimated smooth trend and semi-transparent bands representing the 95% confidence interval. Blue line indicates stress event in Tank 1.
